# Design of a Two-Degree-of-Freedom Mechanical Oscillator for Multidirectional Vibration Energy Harvesting to Power Wireless Sensor Nodes

**DOI:** 10.3390/s24144531

**Published:** 2024-07-13

**Authors:** Hossein Shabanalinezhad, Cesare Svelto, Piero Malcovati, Gianluca Gatti

**Affiliations:** 1 Department of Mechanical, Energy and Management Engineering, University of Calabria, 87036 Rende, Italy; hossein.shabanalinezhad@unical.it; 2 Department of Science, Technology and Society, University School for Advanced Studies Pavia, 27100 Pavia, Italy; 3 Dipartimento di Elettronica, Informazione e Bioingegneria, Politecnico di Milano, 20133 Milano, Italy; cesare.svelto@polimi.it; 4 Dipartimento di Ingegneria Industriale e dell’Informazione, Università degli Studi di Pavia, 27100 Pavia, Italy; piero.malcovati@unipv.it

**Keywords:** piezoelectric energy harvester, spiral beam, wireless sensor, finite element simulation

## Abstract

Converting otherwise wasted kinetic energy present in the environment into usable electrical energy to power wireless sensor nodes, is a green strategy to avoid the use of batteries and wires. Most of the energy harvesters presented in the literature are based on the exploitation of a one-degree-of-freedom arrangement, consisting of a tuned spring-mass system oscillating in the main direction of the exciting vibration source. However, if the direction of excitation changes, the efficiency of the harvester decreases. This paper thus proposes the idea of a curved cantilever beam with a two-degree-of-freedom arrangement, where the two bending natural frequencies of the mechanical resonator are designed to be equal. This is thought to lead to a configuration design that can be used in practical circumstances where excitation varies its direction in the plane. This, in turn, may possibly lead to a more effective energy-harvesting solution to power nodes in a wireless sensor network.

## 1. Introduction

Piezoelectric energy harvesters are innovative devices that capitalize on the piezoelectric effect to convert mechanical energy, such as vibrations or movements, into electrical energy. This ground-breaking technology has gathered considerable attention due to its potential to harvest otherwise wasted mechanical energy from various sources in our environment, ranging from footsteps on pavements to vibrations in machinery.

Piezoelectricity is the capacity of a material to transform mechanical energy into electrical energy without utilizing any additional power [1]. A material with this ability is called a piezoelectric material, that can be divided into two categories of soft and hard types [2]. Piezoelectric materials are mostly flexible, lightweight, highly responsive, and can respond and operate at low excitation frequencies [3,4]. The most common types of piezoelectric material used in an energy harvester are ceramic, polymers, lead-free materials, and nanomaterials. Ceramic piezoelectric materials are commonly used in level sensors, and ultrasonic non-destructive test devices, while piezoelectric nanomaterials are safe for human physiological use. Polymers such as polyvinylidene fluoride (PVDF) have extended life, are environment-friendly (lead-free materials), and consist of organic and inorganic materials [2].

Numerous studies have been conducted to exploit different available sources of energy in the environment such as moving trains [5,6], impact energy from water [7], water flow [8], ocean waves [9], foot movement [10], and other wearable devices [11]. These devices have also been explored as implantable medical devices [12]. Oladapo [13] described how energy harvesters can satisfy the sustainable development goals (SDG) while they are used for the human body. The applications included pericardial nanogenerators real-time monitoring of physiological and pathological signs and aortic piezoelectric energy harvesters.

From the point of view of the energy transduction mechanism, most of the mechanical vibration-based energy harvesters that exist in the literature can be categorized into either piezoelectric, electromagnetic, or electrostatic harvesters [14]. Various studies have been devoted to electromagnetic energy harvesters during the last decade [15,16,17,18]. Electromagnetic harvesting is typically much more robust than piezoelectric harvesting because the latter relies on fragile materials such as ceramics and cannot withstand a large number of load cycles. However, piezoelectric generators are generally more lightweight and compact, and if based on PVDF, they can be reliable and more sensitive to low stresses while generating high-density charge [19,20,21,22,23,24]. Thus, various structures were investigated for piezoelectric generators, such as cantilever beams, circular diaphragms, cymbal transducers, and the stacked array type [25]. The cantilever beam is also utilized in other forms of energy harvesters such as frictional [26] and electromagnetic energy harvesters [27].

Despite all of the benefits of energy harvesters, they still require enhancement in efficiency and practicality. Many researchers have tried to improve the output power and response bandwidth through different approaches such as multi-stability [28,29] and impact generation [30]. In refs. [31,32,33], bi-stability was introduced to increase the efficiency of the harvester. Other studies [34,35] focused on increasing the output power by increasing the peaks in the frequency domain using multiple degrees of freedom systems. A design was proposed in [36] that used extra support along the length of a cantilever beam with an overhang tip mass. In another innovative design, an orthoplanar spring was proposed based on which an electromagnetic energy harvester was manufactured and tested [37]. Other than multi-stability, other types of nonlinearities may be intentionally designed as well as inherently present [38]. Stiffness nonlinearity (e.g., softening and hardening) may be introduced to enlarge the frequency bandwidth [39]. In contrast to a huge portion of the literature where the harvesting devices are adopted in one direction of excitation only, the nonlinear dynamics of a harvester exposed to multidirectional excitation was studied in [40], with a composite beam made of functionally graded materials. In another study [41], a piezoelectric energy harvester robust to multidirectional excitation was studied by attaching the tips of two clamped beams. Biaxial and triaxial vibration energy harvesters were also studied in [42,43], respectively.

Although a great effort has been put into the development and improvement of different types of mechanical vibration-based energy harvesters, the literature is mostly bound to one direction of excitation. The focus of this paper is to design a piezoelectric vibration energy harvester based on a curved cantilever beam with tip mass, vibrating in the horizontal plane (i.e., orthogonal to the direction of gravity), which can be effectively adopted in the case where the direction of the vibrating source is changing within the same horizontal plane. In particular, this study aims at exploring the dynamic performance of a cantilever beam with a spiral shape, which is designed to vibrate in the horizontal plane as a combination of two bending modes with equal natural frequency. The general idea was presented by the authors of this paper in [44], where the analysis was limited to a preliminary finite element simulation only and no design of experiments and experimental validation were reported. The beam is assumed to behave as a linear system, so that a linear dynamic analysis can be performed. A piezoelectric PVDF patch is used in an open-circuit configuration to measure the generated voltage that can be potentially used as an electric power source. This also allowed for validating the fundamental mechanical design, with negligible effect due to electrical current and electro-mechanical coupling. The proposed spiral beam is first studied by finite element analysis using commercial software tools and then manufactured by Fused Deposition Modelling (FDM) 3D printing technique. Experimental tests are finally performed to validate the general design approach and the expected harvesting capability from vibrations in different directions.

## 2. Fundamental Modelling and Simulations

### 2.1. Spiral Beam

**Figure 1:**
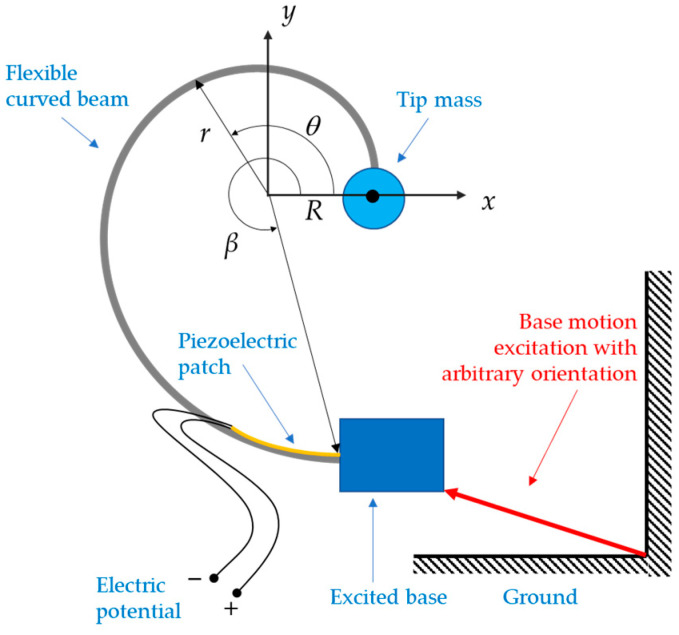
Geometry of the proposed curved beam with tip mass in the *x*–*y* reference frame.

With reference to Figure 1, this paper addresses the case where the curved beam is geometrically defined in the *x*–*y* plane and the vibration occurs in the very same plane, orthogonal to the direction of gravity. The beam is assumed to be excited at its base by an imposed motion in the *x*–*y* plane, with an arbitrary orientation. In contrast to the classical straight cantilever beam design, where the first two bending natural frequencies are far apart from each other [44], here, the spiral shape of the cantilever beam is exploited to explore the case of imposing that the first two bending natural frequencies are equal. This, in turn, will fix some of the design parameters, thus reducing the overall number of the system parameters and focusing on the fundamental concept.

The dynamics of the system is thus studied to investigate its response when the first two fundamentally equal bending natural frequencies are tuned to resonate at the excitation frequency.

A logarithmic spiral beam with polar coordinates (*r*,θ) is specifically proposed that follows Expression (1):

(1) $$r={Re}^{b\theta }$$

where R is the initial radius at θ=0. The parameter *b* > 0 is to be determined along with the specific maximum value β of the angle θ. Figure 1 also shows a schematic of the proposed spiral beam with an ideal point-mass at its tip, excited by an imposed motion at its base with arbitrary orientation.

**Figure 2:**
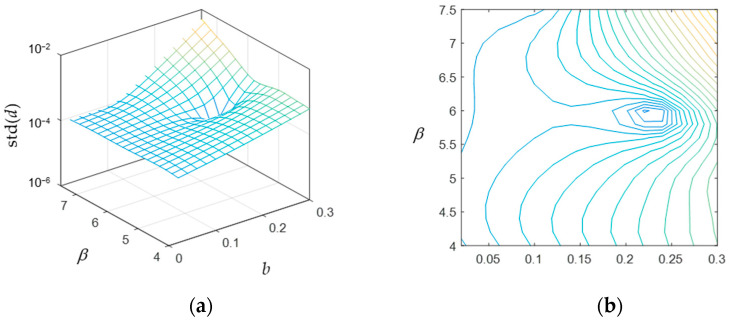
Standard deviation of the tip displacement amplitude (*d*) at different force angles, as a function of the beam geometry (b and β): (**a**) surface plot and (**b**) contour plot.

In the assumption that the cantilever beam is predominantly acting as a stiffness element, with negligible mass relative to the concentrated mass at tip (as in common energy harvesting applications [36,37,45]), to determine the condition where the first two bending natural frequencies are equal, one can design the beam so that its tip has equal stiffness in all directions. To obtain the desired values of *b* and *β* so that the stiffness at the beam tip becomes equal in every direction, an iterative procedure is performed using static analysis in finite element software for given ranges of those two parameters. The beam is thus modelled using Euler–Bernoulli 2D wire elements in the finite element software and grounded at its base. In particular, for each geometry of the beam generated from a specific combination of values of *b* and *β*, a concentrated force is iteratively applied at the beam tip in 8 different directions, each resulting in a particular displacement at the tip. The force orientation angles start at 0° with 45° increments in the same coordinate system where the beam was defined. Since the objective is to impose an equal displacement at the tip, no matter the orientation of the tip force, a circle of displacements at the tip is expected for varying force directions. The standard deviation of each set of displacements associated with a combination of values for *b* and *β* is thus adopted as a metric to compare different geometries of the beam. When the standard deviation of the achieved displacements is very small (compared to its mean value), then it means that the displacement is fairly constant, despite the force orientation at tip. Figure 2 shows the results of the simulations.

**Figure 3:**
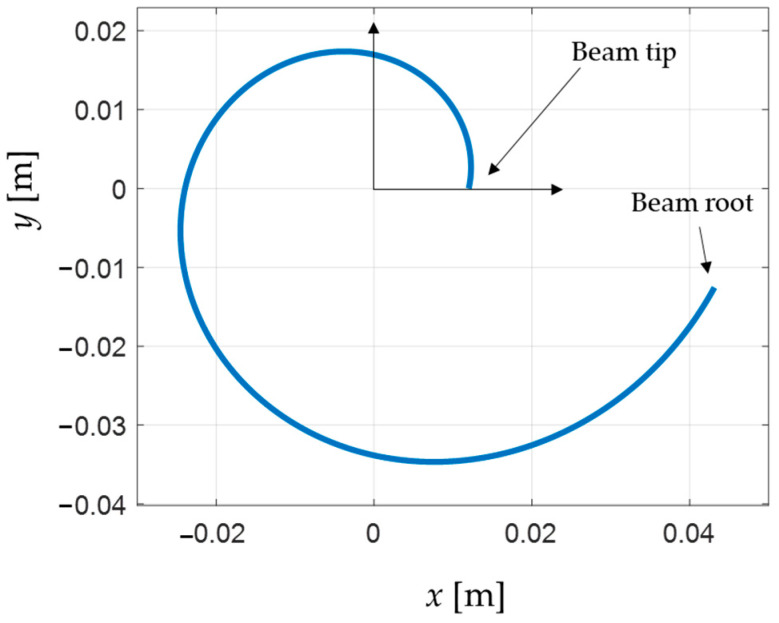
Identified spiral beam shape with equal stiffness at tip.

The result is that the standard deviation of the tip displacement amplitude for varying force direction reaches a minimum for b=0.22 and β=6. This means that the tip displacement amplitude was the most constant for this geometry of the beam over different force angles. Thus, these values are chosen for the spiral beam hereafter in this paper. For practical reasons, mainly related to the manufacturing process and dynamic expectations, the initial radius *R* of the spiral beam was chosen to be 0.012 m, so that the final beam shape is represented in Figure 3.

A finite element simulation is then conducted to study the dynamics of the proposed spiral beam with a concentrated mass at its tip. The beam is modeled in the 3D domain using the beam characteristics that are presented in Table 1. At first, the beam is assumed to be massless (i.e., acting as a stiffness element only), and a 0.02 kg concentrated mass at tip is considered. This specific value for the tip mass is selected to cope with the limitations of the experimental equipment (i.e., the shaker performance). The beam is thus clamped at its base and modal analysis is performed using finite element software with a 10-node quadratic tetrahedron element.

**Figure 4:**
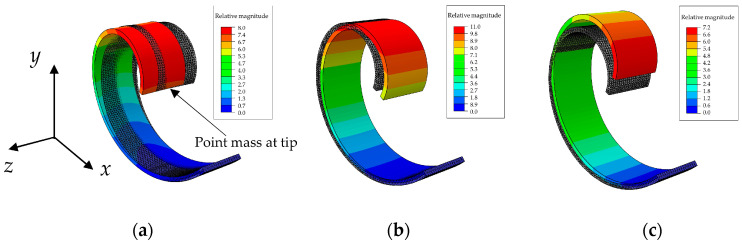
The first 3 mode shapes of the massless beam with point mass at tip: (**a**) mode 1, 22.844 Hz (out-of-plane); (**b**) mode 2, 26.334 Hz (in-plane); and (**c**) mode 3, 26.492 Hz (in-plane).

Figure 4 shows the first three mode shapes of the massless spiral beam with concentrated mass at the tip. The first mode, as depicted in Figure 4a appears to have a different shape and dynamic nature, as it occurs out-of-plane (in the *z* direction orthogonal to the *x*–*y* plane) at a relatively different frequency. Then the two bending mode shapes, i.e., mode 2 in Figure 4b and mode 3 in Figure 4c, possess quite the same natural frequency, although they are along different directions.

In Figure 4, the shaded beam with grey color in all panels is the undeformed beam, while the colorful beam is the one after deformation due to the specific mode shape.

**Figure 5:**
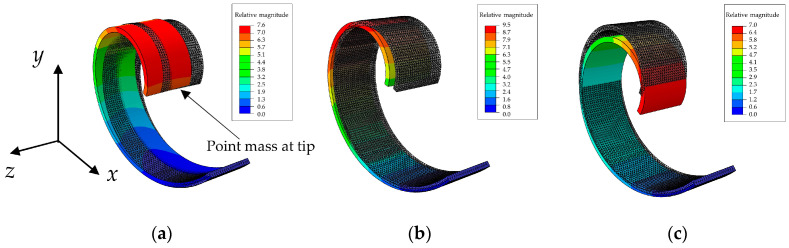
The first 3 mode shapes of the proposed beam with beam density and point mass at tip: (**a**) mode 1, 21.216 Hz (out-of-plane); (**b**) mode 2, 22.319 Hz (in-plane); and (**c**) mode 3, 25.24 Hz (in-plane).

To investigate the effect of the beam density and Poisson’s ratio, simulations using finite element software are also performed in this case, with the parameters listed in Table 1. Notice that the numerical values of these parameters are related to the specific material at hand and used for prototyping, as detailed in Section 3.1 below. Figure 5 shows the simulation results of the first three mode shapes of the proposed spiral beam where the beam is assumed to have mass density, and a point mass is still considered at the tip. As indicated in the figure caption, the difference between the two lowest bending natural frequencies is larger, unlike the case where the beam had no density. All of the simulations and beam parameters are identical to the previous case except for the beam density, which is assumed to have the value presented in Table 1.

As Figure 5 depicts, the two lowest bending natural frequencies are not the same anymore, demonstrating that the beam mass has an effect on the lowest bending frequencies to be equal. This is because the specific orientationally-equal stiffness is designed when the whole mass is concentrated at the beam tip. When the beam has its own mass, then the dynamics of the whole system changes and the lowest two bending natural frequencies are not equal anymore in different directions. However, the higher the mass ratio (tip mass divided by beam mass), the smaller the difference between the lowest bending natural frequencies.

Table 2 compares the natural frequencies of the first three modes, showing the effect of the mass ratio. The relative deviation between the bending natural frequencies is calculated for each mass ratio using Expression (2), which is the ratio of the absolute difference between the lowest two bending natural frequencies divided by their mean value and expressed as a percentage value. This is thus used as a metric to quantitatively show the deviation between two values.

(2) $$Relative\;deviation=\frac{\left|f_{2,\;bending}-f_{1,\;bending}\right|}{\frac{f_{2,\;bending}+f_{1,\;bending}}{2}}\times 100$$

where $f_{i,\;bending}$ (*i* = 1, 2) are the two bending frequencies.

One approach to reduce the effect of the beam mass on the in-plane-bending natural frequencies shift, would be to increase the ratio of the tip mass to the beam mass, as shown in Table 2. This approach, although it may seem relatively easy, comes with some challenges. For instance, a heavier tip mass will lead to a greater static deflection at the beam tip increasing the likelihood of unwanted dynamics to manifest. Clearly, the total weight of the setup will be increased as well, requiring more input energy to induce its motion.

In this research, another approach is thus proposed toward reducing the effect of the beam mass on the difference between the two bending natural frequencies. This is achieved by adding another spiral beam equal to the first one, but in a different assembly arrangement. The second beam can be added by various methods, some of which will be discussed in the next section.

### 2.2. Two-Beam Setup and Configuration

As anticipated in the previous section, one way to reduce the effect of the beam mass on the dynamic response of the system is thought to be achieved by adopting a two-beam setup configuration. The idea is that by adding a second beam equivalent to the first, but with a different arrangement, the effect of the overall mass distribution, with respect to the point mass at tip, is minimized.

It should be noticed that the system natural frequencies will be then generally greater in a two-beam setup configuration than in the single-beam setup configuration, as the equivalent stiffness at the tip would increase twice due to the presence of the two stiffness elements in parallel.

**Figure 6:**
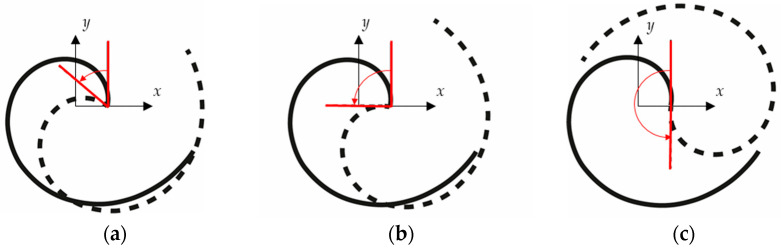
Two-beam setup configuration with different relative angles: (**a**) 50°, (**b**) 90°, and (**c**) 180° inter-beam angle. The spiral beam with solid line is kept fixed, and the other spiral beam with dashed line is rotated.

The main conditions to be satisfied are that the two beams should be in the same plane and be coupled at their tips. This is because they both have an orientationally-equal stiffness at their tips in the *x*–*y* plane, so this property is kept in the new configuration as well. It can be then reasonably deduced that the two beams can be oriented with respect to each other, as shown in Figure 6, which depicts some possible relative orientations of two identical spiral beams.

Simulations using finite element software are conducted to find the best angle between the two beams, called the inter-beam angle hereafter in this paper, to ensure the beam mass effect on the whole system is minimized. One of the two beams is then rotated with respect to the other with a step of 10°. Again, 10-node quadratic tetrahedron elements are used to mesh the beams in the finite element simulations. The beams are clamped at their bases and coupled so that they are free to rotate at their tips, to reduce the in-plane moment at tip and thus keep the modelling formulation discussed for the single-beam setup. As a result of the simulations, the optimal inter-beam angle, to reduce the difference between the two bending frequencies, is found to be 90°. This is speculated to be related to the fact that any tip displacement of the spiral beam in the plane can be described as a linear combination of the first two in-plane bending mode shapes (as long as the excitation frequency is close to these modes, as assumed in [44]), and the fact that the mode shapes are orthogonal by definition.

The results of the simulations leading to the configuration described above are presented in Table 3 for a range of inter-beam angles between 50° to 130°, showing that the three mode frequencies of the beam vary with a change in the relative orientations of the two spiral beams.

**Figure 7:**
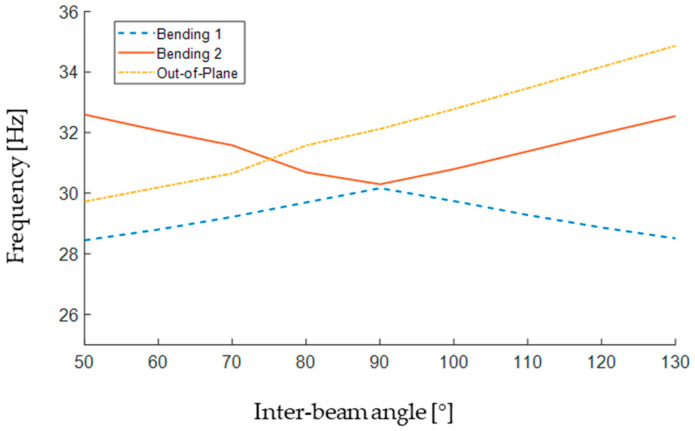
The effect of the inter-beam angle on the frequencies of the first three mode shapes.

One relevant effect of having the two-beam setup is that the mode order is changed based on the angle between the two beams. For instance, Table 3 shows that the out-of-plane mode shape appears as the second for a 50° inter-beam angle, and it appears as the third for an 80° inter-beam angle, whereas it always appears as the first mode in a single-beam setup configuration. The effect of the inter-beam angle on the frequencies of the first three mode shapes is also depicted in Figure 7.

As can be seen in Figure 7, the two bending natural frequencies meet at a 90° inter-beam angle. Moreover, the bending natural frequencies seem to be in a quite symmetric trend (increasing/decreasing in an opposing fashion with the inter-beam angle getting farther from 90°), while the out-of-plane natural frequency has an increasing trend over the whole range of the angle presented in Figure 7. According to Figure 7, the out-of-plane mode jumps to the third position (i.e., the highest frequency) at about a 75° inter-beam angle, making the lower angles less suitable for the setup, as the out-of-plane mode is more likely to interfere with the planar vibration of the beam.

At an inter-beam angle of 90°, the gap between the two bending natural frequencies is at its minimum, while the out-of-plane mode occurs as the third mode, distant enough from the other two frequencies. Needless to say that the tip mass should be attached to the common tip of the two beams in all cases.

It is now clear that the best two-beam configuration, to minimize the beam mass effect on the system dynamics, is to have the second beam rotated by 90° with respect to the first beam. As mentioned above, one main point is to attach the two beams at their tips to achieve orientationally-equal stiffness at the very same tip. Also, as mentioned above, each beam should be allowed to freely rotate about its tip, so that the connection should be by a pin joint.

**Figure 8:**
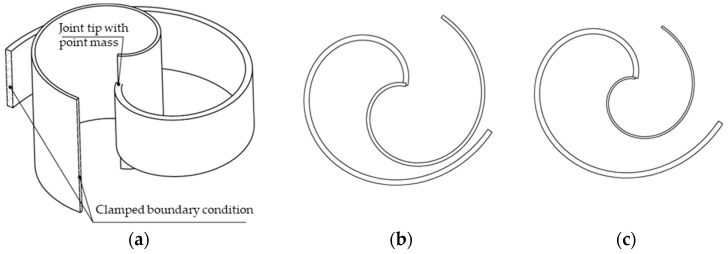
(**a**) CAD model of one beam embedded into the other one. Case where the radius difference of the two beams is (**b**) 20% and (**c**) 40%.

Considering the conditions mentioned above, one possible mechanical assembly arrangement to achieve the desired geometric configuration would be to embed one beam into the other one. However, as depicted in Figure 6b, one can immediately realize that when the two beams are the same, they compenetrate each other. This issue could be solved by reducing the initial radius of the inner beam, thus changing its overall dimensions but keeping the spiral shape. Nevertheless, to keep the stiffness of the smaller beam and its mass constant, one should decrease the thickness of the cross-section and increase the width, so that the total volume of the beam is unchanged, and so its mass. Figure 8a depicts a configuration where the two beams have equal mass and stiffness, but different initial radii. It can be qualitatively seen that the inner beam is thinner and wider than the outer beam.

According to the further schematics shown in Figure 8b,c, if the radius difference of the inner beam is less than about 30% of that of the outer beam, then the gap between the two beams is not large enough to accommodate the vibration displacements of the beams during motion, and the risk for collision between the two beams will be high. Also, if the radius difference is larger than about 30%, then the width of the inner beam will be more than twice that of the outer beam, making the whole setup not well proportionate. Figure 8b,c show two cases where the radius difference between the two beams is 20% and 40%, respectively. It can be qualitatively deduced that the gap between the two beams in Figure 8b is much less than that in Figure 8c, thus introducing a potential risk for collision during working conditions.

**Figure 9:**
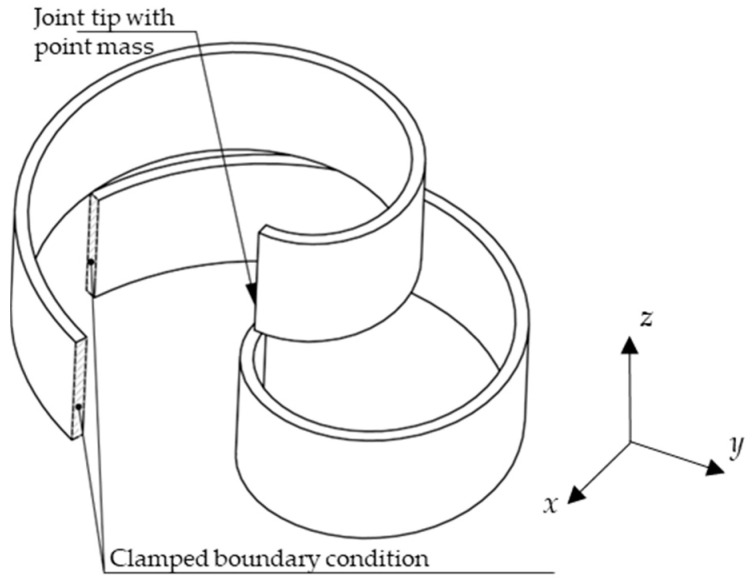
Two identical beams are stacked vertically.

Another way to arrange the two beams is to stack them vertically one at the top of the other. In this case, nominally identical beams can be used, where the suspended mass is also attached to their tips and is located in between the two beams, thus limiting undesired torsional effects and unwanted rotational inertias of the tip mass. Figure 9 depicts a general mechanical assembly arrangement of this configuration.

## 3. Design of the Prototype and Simulations

### 3.1. Details of the Prototype

**Figure 10:**
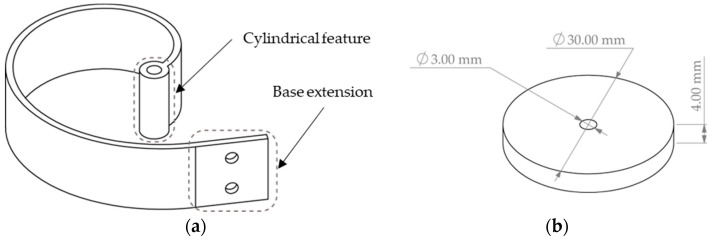
(**a**) Designed beam with cylindrical feature at tip and (**b**) dimensions of the tip mass disc.

Based on the selected two-beam setup configuration and the results of the simulations presented in the previous sections, both beams are designed and manufactured as displayed in Figure 10.

The cylindrical feature at the beam tip illustrated in Figure 10a is added to the model so that the two beams may be assembled and coupled while allowing for a free rotation about the axis of the cylindrical feature. It should be noted that the cylindrical feature axis passes through the beam tip point.

Another practical feature added to the model is the base extension labelled in Figure 10a, which is aimed to be used as a support for attaching the beam to the shaker slip table (i.e., the electro-mechanical exciter) through a beam holder. The base is simply created by extending the beam curve in a straight direction (i.e., tangent to the slope of the spiral) for a length of 20 mm. The two holes depicted in Figure 10a are used for screwing the beam to the beam holder, achieving a fixed boundary condition. Two separate beams, such as the one depicted in Figure 10a, are then 3D-printed. The beam was specifically designed to be manufactured with an FDM 3D printer. The filament used for manufacturing the beam was a Polylactic Acid (PLA) type with the nominal properties presented in Table 1. Given the material properties and the geometrical dimension selected for the beam, the mass of each beam is expected to be 9.47 g, i.e., about half of the tip mass. Since there was no specific need for a high tip mass to beam mass ratio, then a tip mass of about 20 g was kept. It should be noted that keeping the total mass of the setup as low as possible would be beneficial for the feasibility of experiments, due to the limitations of the available electro-mechanical shaker. For this purpose, a small metallic disc made of steel with the nominal dimensions presented in Figure 10b was manufactured and used as the tip mass.

The mass of a disc with the dimensions displayed in Figure 10b and for a nominal material density of 7900 kg/m^3^ would be 22.11 g. Due to manufacturing errors, and real material properties, the final mass of the disc was measured to be 20.6 g.

**Figure 11:**
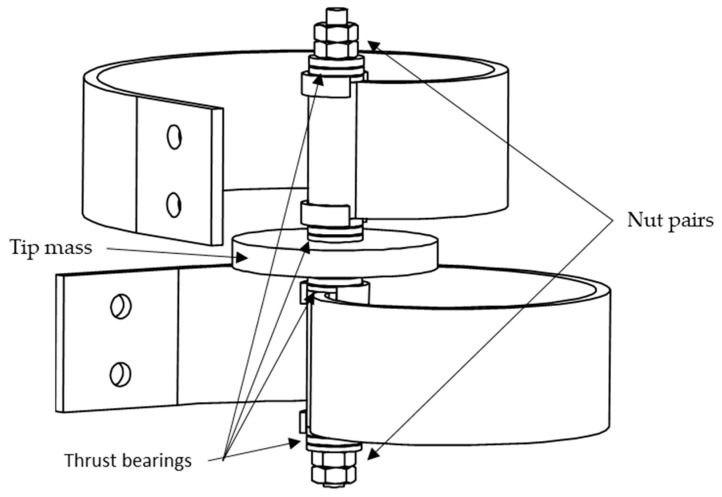
The two-beam configuration assembly.

The two beams were then assembled as shown in Figure 11 using the components presented in Table 4 along with their weights.

As can be seen from Figure 11, the two beams were assembled together at their tips by passing a threaded rod through the cylindrical features of the beams, and the tip mass was held in place using four nuts in total. In addition, four thrust bearings were also included in between the components, to guarantee the smooth rotation of all parts, reducing friction. Since thrust bearings are susceptible to the axial force of fasteners, a pair of nuts was used at each end ensuring self-locking and limiting loosening of tightness due to vibration. Also, a fixed axial clearance was maintained between all components to allow correct motion.

**Figure 12:**
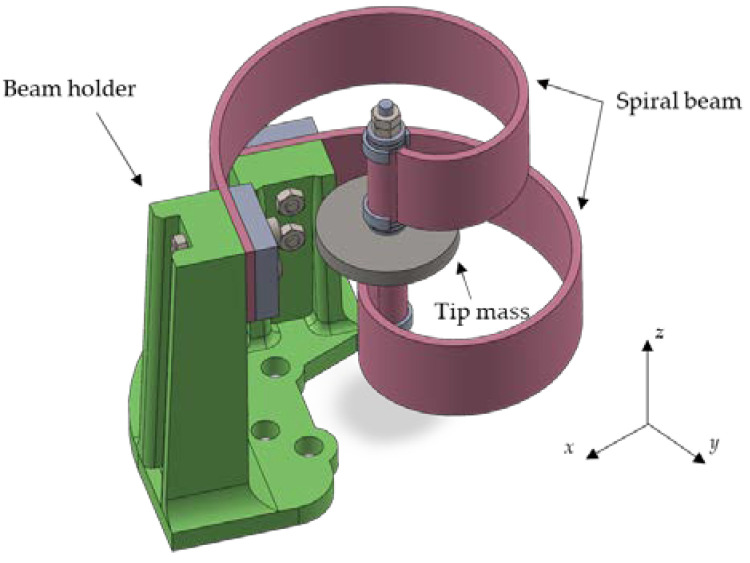
3D model of the experiment setup.

Finally, both beams were fixed to the beam holder using screws, as better depicted in Figure 12, which also provides some further details of the mechanical assembly.

Since the beam holder was of the same material as the beams, it was numerically studied using finite element analysis to ensure it did not interfere with the beam vibrations. In particular, the lowest natural frequency of the beam holder is greater than 450 Hz, which is much larger than the two first bending natural frequencies (around 20–30 Hz) of the two-beam setup.

### 3.2. Detailed Finite Element Validation

Before manufacturing the components for the experiment setup, more detailed finite element simulations aree run to investigate the setup more thoroughly. In this set of simulations, the beam model contains, unlike the previous simulations, the cylindrical feature at the tip to mimic the real-world component behaviors. The tip mass is simulated as a point mass, as its rotational inertia effect is considerably reduced in the real setup since the tip mass can freely rotate about its longitudinal axis. The real properties of the PLA material after 3D-printing were also measured on a simple specimen, to avoid using nominal values as in Table 1, which would sensibly affect the expected theoretical results. The Young’s modulus was then measured to be 2.8 GPa from a simple bending test on a straight cantilever beam arrangement, and the mass density was measured to be 1250 kg/m^3^.

**Figure 13:**
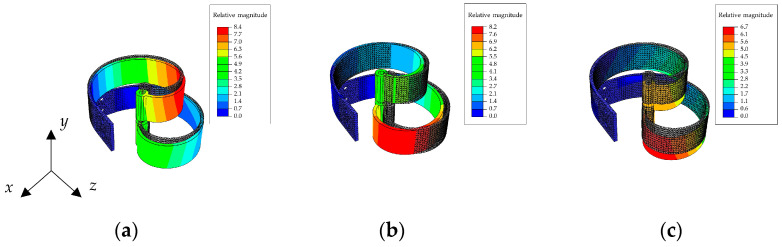
The first 3 mode shapes of the detailed beam: (**a**) mode 1, 23.688 Hz (in-plane); (**b**) mode 2, 23.795 Hz (in-plane); and (**c**) mode 3, 24.925 Hz (out-of-plane).

Figure 13 shows the simulation results. The first two modes are of bending type with quite equal natural frequencies (i.e., 23.688 Hz and 23.795 Hz) and the third mode is out-of-plane with a relatively distant natural frequency (24.925 Hz). It should be noted that the tip mass in this simulation has the value presented in Table 4, so these results are in line with those obtained in the experiment.

The grey beam model is again the undeformed one, while the colorful models are the beams deformed due to each mode shape. As expected, finite element simulations confirmthe reduction of the beam mass effect on the bending natural frequencies in a setup consisting of two equal beams. Moreover, the presence of a relatively low tip mass to beam mass ratio, which is about 2.9 in this setup, was shown to be ineffective, as expected by the preliminary analysis.

**Figure 14:**
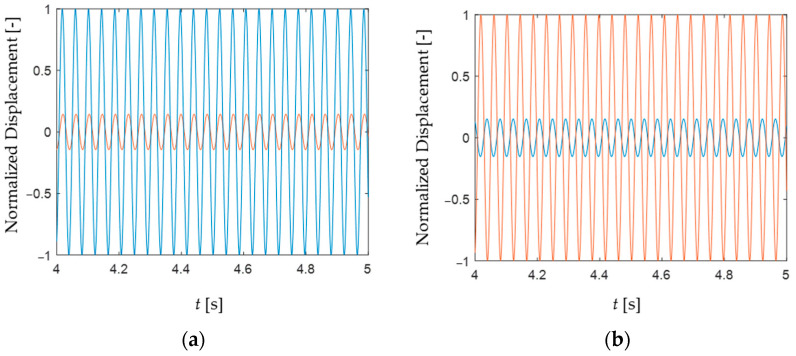
Dynamic analysis of the beams tip, showing the normalized tip displacement at steady-state (i.e., after 4 s) for a base excitation of 0.2 mm along (**a**) the *x* direction and (**b**) the *y* direction. The blue line is the response along the *x* direction, and the red line is the response along the *y* direction.

A dynamic analysis of the setup is also conducted to further study the behavior of the system before manufacturing it. In these new simulations, the base of the two beams is excited with a relatively low displacement amplitude (0.2 mm) harmonic motion, in order not to excite any nonlinearity due to large deflections. The excitation is applied, at the bending resonance frequency of the setup, in one direction only, first along the *x* direction and then along the *y* direction. The tip displacement time histories, along the *x* and *y* directions, are extracted after the occurrence of a transient (lasting about 4 s) due to the initial conditions. These time histories are then normalized to the maximum value of each test, so that their relative difference could be better appreciated, independently from the mechanical damping value used (if relatively low), which could not be estimated from the experimental setup. For instance, using a 2% structural damping value in the simulation, leads to a maximum displacement amplitude at the tip of about 8.5 mm. Figure 14 thus shows the steady-state response of the setup for the two different excitation directions mentioned above.

The dynamic analysis reveals that the beam tip moves with two components in the plane, even when the excitation is in one direction only. Since the two bending modes have quite the same natural frequency and the excitation direction is not specifically in the direction of any of these modes, then it is very likely that both modes are being activated. However, the mode which is oriented closer to the excitation direction is excited more and gets the highest amplitude, absorbing a larger portion of the total input energy.

## 4. Experimental Work

**Figure 15:**
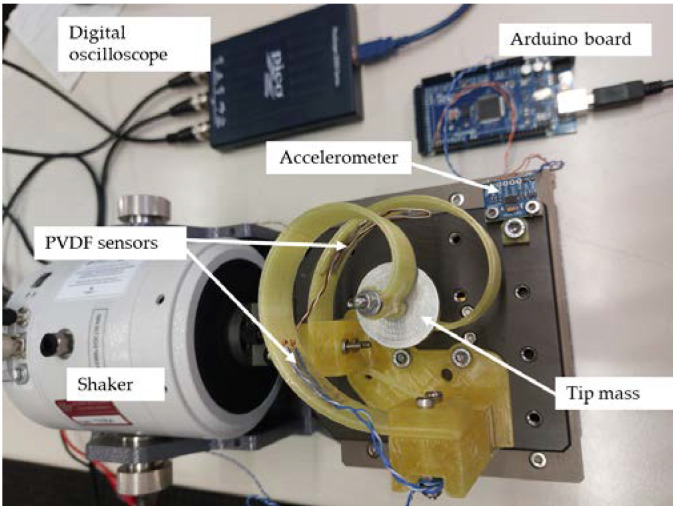
Test setup with components and equipment as labelled.

After running the simulations and validating the design, all of the components were manufactured using an FDM 3D printer with a PLA filament. The infill density was set to 100% for all components so their mechanical properties would be closer to the nominal values. Figure 15 shows a photo of the final assembled setup consisting of two identical beams, for experimental testing.

The whole setup was excited using a 9363-EM-2F electro-dynamic mini-shaker by Sentek dynamics (Santa Clara-CA, USA). The rated peak force of the shaker for a sine wave excitation was 20 N with the main natural frequency of 17 kHz. The frequency range of the shaker was thus 0.3 Hz–15 kHz, with a maximum peak-to-peak displacement and velocity of 5 mm and 0.1 m/s, respectively. The shaker also had a built-in amplifier which made it usable with as low as 2 volts of input signal.

A 2204A USB oscilloscope by PICO Technology (St Neots, UK) was used to acquire the vibration data through a personal computer. The sampling rate of the oscilloscope was 100 MSa/s and the bandwidth was 10 MHz. The digital oscilloscope also had an embedded signal generator which was used to generate a sine wave as the input signal to the shaker. The function generator could produce waves up to 100 kHz with a maximum voltage amplitude of 800 mV. As depicted in Figure 15, an ADXL345 accelerometer by Analog Devices (Wilmington-MA, USA) was attached to the slip table, so the excitation acceleration was acquired with the help of a 2560 Mega board by Arduino (Monza, IT) through the same personal computer. The maximum sampling frequency of the accelerometer was 1600 Hz, which was suitable for the frequency range of the experiments. The serial input pins of the Arduino board were used to connect the accelerometer sensor.

The acceleration amplitude, α (m/s^2^), for a sinusoidal excitation with displacement amplitude, δ (m), at frequency, *f* (Hz), can be expressed as follows:

(3) $$\alpha =\delta {\left(2\pi f\right)}^{2}$$

Since the simulations reveal the setup bending natural frequencies to be about 23.8 Hz, the tests were conducted for a few discrete frequencies around this frequency value, to find the real natural frequency of the setup and study its dynamic behavior at resonance. Table 5 presents the required base acceleration for each selected natural frequency to reach a displacement amplitude of 0.2 mm, as from Equation (3).

The selection of the displacement amplitude adopted for the test was motivated in order not to cause any risk of collision among the system components during operation. For the setup at hand, the tip displacement should be kept below 10 mm at resonance, to limit such a risk.

The vibration of each beam was then investigated using the open-circuit voltage produced by two 13 by 25 mm PVDF sensors attached to the beams. This is used in this preliminary work to validate the initial design which did not take into account any electro-mechanical coupling with the piezoelectric patch. The voltage produced by each sensor was then collected through the digital oscilloscope and a personal computer. The sensors were attached to the same location on both beams. Figure 15 shows the PVDF sensor locations on the beams, along with other experimental hardware, also including the digital oscilloscope.

**Figure 16:**
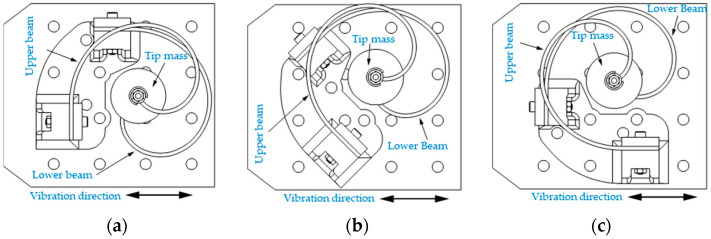
Three different relative excitation orientations: (**a**) 0°, (**b**) 45°, and (**c**) 90°.

The tests were conducted for three excitation directions and for each frequency presented in Table 5, which made a total number of 21 tests. The change in excitation direction was achieved by rotating the beam holder with respect to the exciting shaker, which had a fixed direction of shaking. The orientations were 0°, 45°, and 90° and the definition of each angle is graphically described in Figure 16.

It should be noted that since the two beams are in a 90° configuration with respect to each other, the 0° and the 90° orientations of the excitation are expected to produce nearly the same total amount of energy. The same applies to a 45° compared to a 135° orientation.

## 5. Results and Discussion

**Figure 17:**
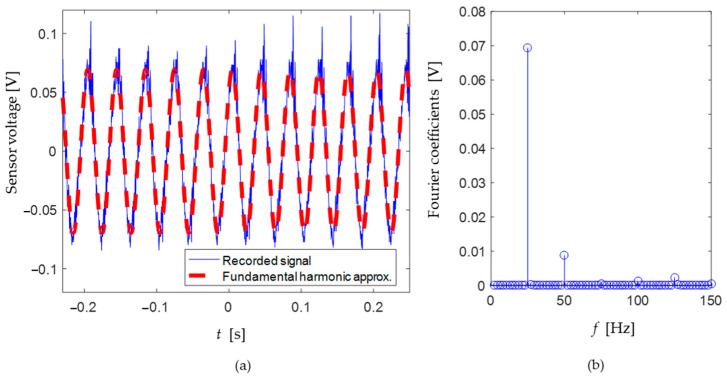
(**a**) PVDF sensor voltage output as a function of time when the excitation was at resonance (25 Hz) and the relative orientation of the setup was 90°. (**b**) Corresponding Fourier coefficients.

The output voltage was acquired by the digital oscilloscope that simultaneously calculates the waveform root mean square (RMS) value. Thus, all of the voltage measurement results presented in this paper were obtained from this device. Figure 17a shows the recorded signal from the digital oscilloscope when the excitation was at resonance (25 Hz) and the relative orientation of the setup was 90°. Figure 17b shows the Fourier coefficients of the signal in Figure 17a. It can be seen that the system response is predominantly harmonic at the excitation frequency, and the amplitude of higher harmonics is negligible.

**Figure 18:**
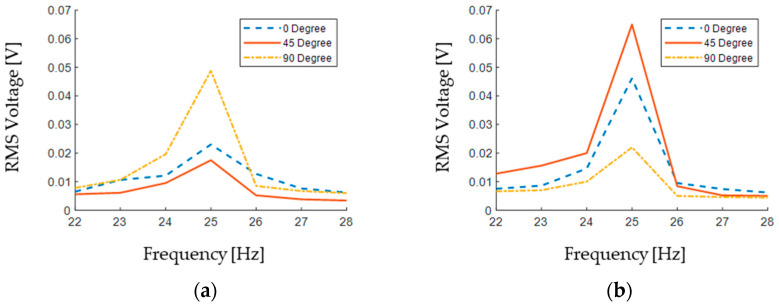
Output voltage of the PVDF sensors: (**a**) upper beam and (**b**) lower beam.

Figure 18 compares the output RMS voltage of the two PVDF patches attached to the upper and lower beam, for seven different excitation frequencies and three orientations.

The test results revealed that the natural frequency of the setup was about 25 Hz and that the system remained in its linear domain. This should be compared to the average bending natural frequency from the finite element simulations, which was about 24 Hz. The excitation directions considered in the experiments had no effect on the system response, proving that the system is robust to the excitation direction.

However, the strain distribution along the beam length is affected by the excitation orientation, which is the reason for the different generated voltage in Figure 18. 

Since there is a 90° angle between the two beams, one could expect the results of the lower beam to be equal to those of the upper beam when the setup is rotated 90°. The obtained results prove this. For instance, the maximum RMS value of the output voltage of the upper beam in the 0° rotation is 0.023 V (blue dashed line in Figure 18a), and this is quite similar to that of the lower beam in the 90° setup rotation (yellow dash-dotted line in Figure 18b), which is 0.022 V.

The output RMS voltages for the 45° rotation of the setup in Figure 18a,b are, however, different between the upper and lower beams. That is simply because when the upper beam is in the 45° orientation, then the lower beam is rotated 135°.

**Figure 19:**
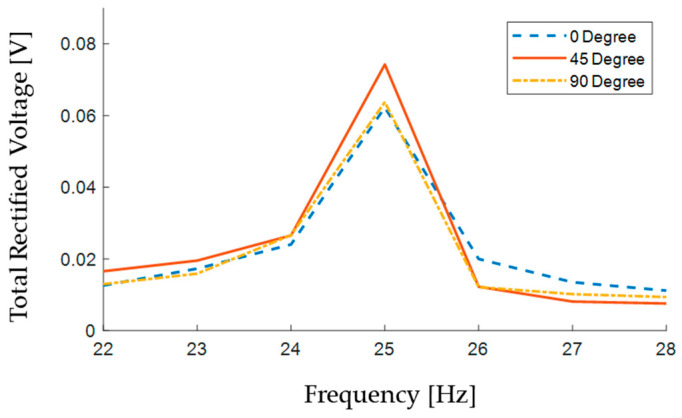
Sum of the two ideally rectified open-circuit voltages.

Assuming the two generated AC voltages undergo a full-wave rectification to DC, they will ideally result in V_DC_ ≈ 0.9V_RMS_. The two piezoelectric patches could then be connected in series to charge a capacitor for on-demand use (i.e., for acquiring sensor data and wireless data transmission). To preliminarily verify the overall performance of the coupled PVDF design at different excitation directions, the AC–DC converted voltages of the two patches are added together to obtain an estimate of the total produced DC voltage. This is plotted in Figure 19, and it can be seen that the estimate for the total generated voltage does not vary much with respect to the excitation direction.

It is worth noticing that the open-circuit voltage collected from the experimental setup at hand was relatively low, as the optimization of the system for power generation was out of the scope of this present work, which was aimed at validating the fundamental idea of the mechanical oscillator. In case of such a low-amplitude voltage application, full-wave rectifiers may cause unacceptable voltage drops on the diodes, and different energy conversion strategies could then be adopted, such as synchronous rectification, based on MOSFET technology [46].

## 6. Conclusions

This paper has investigated the feasibility of using spiral beams in a cantilever configuration with a tip mass, for the benefit of harvesting energy from environmental vibrations and generating electrical voltage to supply sensor nodes, without the need for wirings and disposable batteries. The main assumption was that the excitation was in the plane, with constant frequency but varying direction. The parameters of the spiral beam were obtained by the numerical iteration of a series of finite element simulations trying to achieve an equal stiffness at the beam tip, thus making the first two in-plane bending natural frequencies coincident.

Practical issues have been considered, such as the out-of-plane motion and the ratio of the tip mass to that of the beam. A configuration of two identical spiral beams was proposed to overcome the above-mentioned issues. Compared to the single spiral beam, the two-beam setup configuration allowed for a lower tip-to-beam mass ratio by reducing the effect of the beam mass on the fundamental bending frequencies and eliminating the need for a relatively heavy tip mass. In addition, a relatively lighter tip mass reduces the total weight of the setup, lowering the required input energy to excite the oscillator. The optimum angle between the two beams assembled in a stacked arrangement was obtained based on several finite element simulations, minimizing the difference between the two bending natural frequencies, and it turned out to be 90°.

A test setup was finally designed and manufactured based on the results of several finite element simulations. The experiments were conducted using an electro-dynamic shaker as an exciter, one PVDF sensor attached to each beam, an accelerometer sensor at the excited base, and a digital oscilloscope to measure the piezoelectrically generated voltages. The PVDF sensors were used to measure the open-circuit voltage generated due to the input vibration in different directions.

A total number of 21 tests were conducted for different combinations of frequency values and excitation directions. Assuming an ideal full-wave rectification of the piezoelectrically generated voltages to DC, an estimated trend of the total generated open-circuit voltage produced by the two-beam setup in each test was calculated. According to these results, the whole system configuration is potentially able to generate a total open-circuit voltage which varies slightly with changes in the excitation direction.

The analysis presented in this paper shows that the conceptual idea of using a curved beam with an equal equivalent stiffness at its tip in all directions is an interesting solution to be further investigated for designing vibration energy harvesting devices. Potential issues may come from the strain distribution along the beam, which may involve an optimization of the sensor location. Future developments will also address issues related to electro-mechanical coupling, electrical load effect, and generated power.

## Figures and Tables

**Table 1:** The curved beam characteristics.

Young’s Modulus [GPa]	Density [kg/m^3^]	Poisson’s Ratio	Tip Mass [kg]
3.5	1240	0.33	0.02

**Table 2:** The first three natural frequencies of a single beam for different mass ratios.

Mass Ratio	Mode 1 [Hz]	Mode 2 [Hz]	Mode 3 [Hz]	Relative Deviation [%]
∞	22.844 *	26.334	26.492	0.60
10	11.350 *	12.746	13.247	3.85
5	15.747 *	17.254	18.503	6.99
2	23.567 *	24.286	28.231	15.02
1	29.432	30.669 *	37.787	24.86

* Out-of-plane mode shape.

**Table 3:** Three first natural frequencies for various inter-beam angles.

Rotation Angle [°]	50	60	70	80	90	100	110	120	130
Mode 1 [Hz]	28.447	28.802	29.22	29.696	30.167	29.741	29.281	28.867	28.508
Mode 2 [Hz]	29.725 *	30.192 *	30.654 *	30.691	30.296	30.796	31.382	31.973	32.544
Mode 3 [Hz]	32.596	32.066	31.582	31.577 *	32.121 *	32.776 *	33.467 *	34.172 *	34.864 *

* Out-of-plane mode shape.

**Table 4:** Mass of components.

Part Name	Mass [g]
Nut X4	0.91
Thrust bearing X4	3.42
Threaded rod (0.07 m)	2.62
Disc	20.6
Total tip mass	27.55

**Table 5:** Base acceleration required for a 0.2 mm base displacement amplitude at each frequency.

Frequency [Hz]	22	23	24	25	26	27	28
Acceleration [m/s^2^]	3.82	4.17	4.55	4.93	5.34	5.76	6.19

## Data Availability

The data that support the findings of this study are available from the corresponding author upon reasonable request.
